# Correlation of retinal thickness, macular volume, and their fluctuation with visual outcomes in patients with macular edema due to retinal vein occlusion

**DOI:** 10.1186/s40942-025-00693-2

**Published:** 2025-06-16

**Authors:** Kwanchanok Rattanalert, Patama Bhurayanontachai, Mansing Ratanasukon, Pichai Jirarattanasopa, Wantanee Dangboon Tsutsumi

**Affiliations:** Department of Ophthalmology, Faculty of Medicine, Prince of Songkla University, Songkhla, Thailand

**Keywords:** Retinal vein occlusion, Macular edema, Central retinal thickness, Macular volume, Anti-vascular endothelial growth factor (anti-VEGF)

## Abstract

**Background:**

This study aimed to examine the utility of macular volume (MV), central retinal thickness (CRT), and their fluctuations for predicting post-treatment visual acuity in patients with macular edema (ME) secondary to retinal vein occlusion (RVO).

**Methods:**

This retrospective cohort study included patients treated with intravitreal anti-vascular endothelial growth factor (anti-VEGF) therapy for ME due to RVO at a tertiary university hospital between August 2016 and July 2020. We identify the correlation of the MV, CRT, with their fluctuations, and best-corrected visual acuity (BCVA) measured using optical coherence tomography at baseline and at 1-, 3-, 6-, and 12-months post-treatment.

**Results:**

Among the 74 eyes included, 27 and 47 had central RVO (CRVO) and branch RVO (BRVO), respectively. Following anti-VEGF therapy both, the CRVO and BRVO group exhibited significant improvements in BCVA, CRT, and MV compared to baseline. In all patients, MV was consistently correlated with BCVA, whereas CRT was correlated with BCVA at selected time points. In patients with CRVO, MV was a better predictor of post-treatment visual outcomes than CRT. Moreover, fluctuations in CRT and MV correlated with BCVA over 12 months.

**Conclusions:**

MV yielded more correlation with visual outcomes in patients with RVO and ME receiving anti-VEGF therapy than CRT. Considering concurrent MV and CRT measurements could enhance more precision of treatment assessment, especially in CRVO patients.

## Introduction

Retinal vein occlusion (RVO) is the second most common retinal vascular disease. It predominantly affects middle-aged individuals, and the incidence increases more than ten-fold between the ages of 40 and 65 years [1]. RVO manifests as two distinct types: branch RVO (BRVO) and central RVO (CRVO). Hemiretinal vein occlusion (HRVO) is considered a subtype of BRVO. Macular edema (ME) is the leading cause of vision loss in RVO, with 21% of patients having visual acuity ≤ 20/50 [2]. Intravitreal injection of anti-vascular endothelial growth factor (anti-VEGF) agents, which is the first-line therapy, effectively alleviates ME with a favorable safety profile [3–5].

Optical coherence tomography (OCT) is the primary imaging modality for assessing RVO-related ME. Central retinal thickness (CRT) is a key OCT parameter that is used to determine disease activity and progression and treatment response. Increased CRT is associated with loss of function, and vice versa. However, ME can affect the parafoveal area before the foveal region, and its resolution may begin in the parafoveal area before the central fovea. Few reports leveraged MV as a factor in treatment decision. Prior researches have identified CRT and macular volume (MV) as predictive factors for visual outcomes after anti-VEGF therapy for RVO [6–8]. Post-hoc analyses in the COPERNICUS, GALILEO, and VIBRANT studies revealed weak-to-moderate and moderate associations between CRT and visual outcomes at baseline in eyes with ME due to CRVO and BRVO, respectively [9]. Although reductions in MV and CRT are correlated with improvements in final visual acuity (VA) over time, the usefulness of CRT as a reliable marker of visual outcomes has been questioned [7]. Furthermore, macular changes may predict visual outcomes in patients with RVO treated with anti-VEGF agents [10].

Considering the importance of these factors in evaluating disease progression and treatment response, this study aimed to compare the usefulness of the OCT parameters CRT and MV and their fluctuations for predicting post-treatment VA in patients with RVO and ME.

## Materials and methods

### Patients and study design

We retrospectively reviewed the medical records of patients with RVO complicated by ME, who were treated with intravitreal injection of anti-VEGF agents, including bevacizumab, ranibizumab, and aflibercept, at Songklanagarind Hospital, a tertiary university hospital, between August 2016 and July 2020. The study adhered to the principles outlined in the Declaration of Helsinki and was approved by the Institutional Review Board of Faculty of Medicine, Prince of Songkla University (REC. 64-035-2-4; 22 February 2021). The data extraction and data analysis processes were conducted, starting from 24 February 2021, in accordance with the Personal Data Protection Act. As deidentified data were used, with minimal or no risk to the patients, the requirement for informed consent was waived.

The inclusion criteria were diagnosis of CRVO, BRVO, or HRVO according to the International Classification of Diseases, Tenth Revision (ICD-10) codes H34.8 (other retinal vascular occlusions) and H34.9 (retinal vascular occlusion unspecified). ME was defined as CRT > 250 μm with presence of retinal fluid on spectral-domain OCT images, regardless of other internal structural changes. All OCT images were obtained by using the Spectralis^®^ system (Heidelberg Engineering, Heidelberg, Germany). All patients received intravitreal injection of an anti-VEGF agent at month 1 and in pro-re-nata regimen from month 2 onwards. The scheduled visits were every 4–5 weeks during the first three months and every 6–8 weeks thereafter, or every 4–5 weeks if macular edema persisted. Switch of anti-VEGF agents was allowed in non-responder. Patient who was switched to steroid or laser treatment would be excluded from the study. Patients with HRVO were categorized into the BRVO group. The exclusion criteria were incomplete baseline characteristic data; follow-up time < 12 months; and switching to alternative treatments such as intravitreal injection of steroids, laser therapy, or surgical interventions.

### Data collection

We recorded patients’ demographic characteristics; best corrected VA (BCVA); and OCT measurements, including CRT, MV, and their standard deviations (SDs), at baseline and 1, 3, 6, and 12 months after the initial intravitreal injection of anti-VEGF agents. All OCT scans captured picture of the macula with ETDRS grid being centered on the fovea. The signal strength should be at least 6/10. CRT was defined as the average thickness within the central 1-mm circle of the retinal thickness map. MV was defined as the total retinal volume within the central circular area measuring 6 mm in diameter. BCVA was quantified using Early Treatment Diabetic Retinopathy Study visual acuity charts and converted to the logarithm of the minimal angle of resolution (logMAR) units for statistical analysis.

### Statistical analysis

Demographic data were summarized based on variable type. The distribution of continuous variables was assessed using the Kolmogorov–Smirnov test to determine conformity to the normal distribution. Normally distributed continuous variables were presented as mean ± standard deviation (SD), whereas non-normally distributed variables were expressed as median with interquartile range (IQR). Categorical variables were reported as absolute frequencies and percentages. Comparative analyses between patients with CRVO and BRVO were performed using the independent t-test for normally distributed continuous variables, the Mann–Whitney U test for non-normally distributed continuous variables, and either the Chi-square test or Fisher’s exact test for categorical variables, as appropriate based on cell counts.

Pearson’s correlation coefficient (r) was employed to assess the strength and direction of linear associations between CRT, MV, and BCVA across multiple time points. The interpretation of correlation strength followed standard classification criteria as defined in the literature [11].

A linear regression model was employed to evaluate the predictive and potentially causal relationship between BCVA at 12 months and the standard deviations (SD) of CRT and MV. The strength of the association was quantified using the regression coefficient (B) along with its 95% confidence interval (CI), and model fit was assessed using the coefficient of determination (R²). A *p*-value of less than 0.05 was considered indicative of statistical significance.

Statistical analyses were conducted using MedCalc Statistical Software version 22.017 (MedCalc Software Ltd., Ostend, Belgium; https://www.medcalc.org; 2024) and IBM SPSS Statistics for Macintosh, version 27.0 (IBM Corp., Armonk, NY, USA).

## Results

### Baseline characteristics

**Fig. 1:**
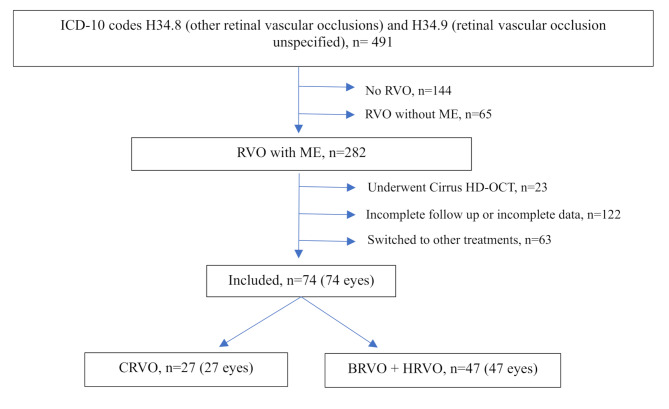
Flowchart of the patient selection process

A total of 491 patients were identified using the ICD-10 codes for RVO. Of these, 74 eyes from 74 patients met the inclusion criteria; 27 (36.5%) and 47 (63.5%) eyes were diagnosed with CRVO and BRVO (including HRVO), respectively (Fig. 1). The remaining 417 patients were diagnosed with other vascular conditions such as retinal artery occlusion, ocular ischemic syndrome, proliferative diabetic retinopathy, age-related macular degeneration, carotid cavernous fistula, and vitreous hemorrhage. Of the 491 patients, 65 patients who did not exhibit ME according to the specified criteria and 23 patients diagnosed with ME using a different imaging modality were excluded. Additionally, 122 patients who did not complete the 12-month follow-up or had incomplete data, and 63 who patients were switched to alternative treatments, including 45 who underwent panretinal laser therapy, four who received intravitreal triamcinolone, 11 who received intravitreal dexamethasone implants, and three who underwent pars plana vitrectomy, were excluded.

ICD-10, International Classification of Diseases, Tenth Revision; RVO, retinal vein occlusion; ME, macular edema; CRVO, central retinal vein occlusion; BRVO, branch retinal vein occlusion; HRVO, hemiretinal vein occlusion.

Table 1 presents the baseline patient demographic and ocular characteristics. Other systemic underlying diseases comprised chronic kidney disease, Parkinson disease, endometrium carcinoma, lymphoma, and hyperthyroidism. Ocular comorbidity included glaucoma. The demographic characteristics did not differ between groups.

**Table 1:** Baseline characteristics

	Overall RVOs (*n* = 74)	CRVO (*n* = 27)	BRVO (*n* = 47)	*P* Value
Age at first diagnosis (years)				
Mean ± SD Median (IQR)	60.93 ± 11.63 63.78 (53.35, 68.27)	60.43 ± 11.29 61.90 (51.96, 67.87)	61.23 ± 11.93 63.89 (53.49, 69.29)	0.78 0.67
Sex, No. (%)				
Male Female	33 (44.6) 41 (55.4)	16 (59.3) 11 (40.7)	17 (36.2) 30 (63.8)	0.06
Laterality, No. (%)				
Unilateral	74 (100)	27 (100)	47 (100)	-
Eye, No. (%)				
Right Left	36 (48.6) 38 (51.4)	13 (48.2) 14 (51.8)	23 (48.9) 24 (51.1)	0.95
Underlying systemic disease, No. (%)	50 (67.6)	20 (74.1)	30 (63.8)	0.37
Diabetic mellitus	17 (23.0)	8 (29.6)	9 (19.1)	0.30
Hypertension	36 (48.6)	16 (59.3)	20 (42.5)	0.17
Dyslipidemia	25 (33.8)	9 (33.3)	16 (34.0)	0.95
Cardiovascular disease	5 (6.8)	1 (3.7)	4 (8.5)	0.65
Others	7 (9.5)	2 (7.4)	5 (10.6)	1.00
Ocular comorbidity, No. (%)	4 (5.4)	3 (11.1)	1 (2.1)	0.14
BCVA (LogMAR)				
Mean ± SD Median (IQR)	0.79 ± 0.61 0.61 (0.32, 0.91)	1.04 ± 0.73 0.7 (0.41, 1.6)	0.64 ± 0.48 0.59 (0.3, 0.8)	0.01* 0.02*
IOP (mmHg)				
Mean ± SD Median (IQR)	14.01 ± 3.70 13 (11, 16)	14.26 ± 4.41 13 (11, 17)	13.86 ± 3.26 14 (11, 16)	0.66 0.93
CRT (µm)				
Mean ± SD Median (IQR)	584.0 ± 206.80 538 (446, 717)	688.52 ± 233.53 668 (497, 879)	523.96 ± 164.16 524 (395, 595)	< 0.01* < 0.01*
MV (mm^3^)				
Mean ± SD Median (IQR)	12.11 ± 2.46 11.8 (10.23, 13.41)	13.25 ± 2.83 12.29 (11.08, 14.97)	11.46 ± 1.96 11.22 (9.82, 13.04)	< 0.01* < 0.01*
Total number of injections				
Mean ± SD Median (IQR)	4.42 ± 2.21 4 (3, 5)	4.71 ± 1.90 5 (3, 5.5)	4.23 ± 2.37 4 (3, 5)	0.37 0.22

CRVO, central retinal vein occlusion; BRVO, branch retinal vein occlusion; BCVA, best-corrected visual acuity; SD, standard deviation; logMAR, logarithm of the minimal angle of resolution; IOP, intraocular pressure; CRT, central retinal thickness, MV, macular volume

*Significant *P*-value

The prevalence of specific conditions, such as diabetes mellitus, hypertension, dyslipidemia, cardiovascular disease, and other systemic diseases, was not significantly different between the CRVO and BRVO groups. Underlying ocular diseases were present in 5.4% of patients with RVO, including 11.1% and 2.1% of patients in the CRVO and BRVO groups, respectively (*P* = 0.14). The baseline BCVA was significantly worse in the CRVO group compared to that in the BRVO group (1.04 ± 0.73 logMAR vs. 0.64 ± 0.48 logMAR; *P* = 0.01). Intraocular pressure by air-puff tonometry was not significantly different between the groups, with an overall mean of 14.01 ± 3.70 mmHg (*P* = 0.66). However, CRT and MV were significantly greater in the CRVO group than in the BRVO group (CRT, 688.52 ± 233.53 μm vs. 523.96 ± 164.16 μm, *P* < 0.01; MV, 13.25 ± 2.83 mm³ vs. 11.46 ± 1.96 mm³, *P* < 0.01).

The mean number of anti-VEGF injections at six months and one year in CRVO group were 3.32 ± 1.02 (min 2, max 6) and 4.71 ± 1.90 (min 2, max 9), respectively. The mean number of anti-VEGF injections at six months and one year in BRVO group were 3.06 ± 1.36 (min 0, max 6) and 4.24 ± 2.38 (min 1, max 11), respectively. No statistical difference in number of injections between groups in both time frames.

### Longitudinal changes of vision and OCT parameters after anti-VEGF treatment

Table 2 demonstrates the longitudinal changes of vision and OCT parameters during anti-VEGF treatment.

#### Visual acuity (BCVA)

At baseline, mean BCVA was significantly worse in the CRVO group compared to the BRVO group. Both subgroups exhibited significant visual improvement over time (all pairwise comparisons vs. baseline, *p* < 0.001), although between-group differences diminished at subsequent time points and did not reach statistical significance after the first month.

#### Central retinal thickness (CRT)

At baseline, CRT was markedly higher in CRVO than in BRVO eyes. Both groups exhibited substantial CRT reductions after treatment, with the most significant decline observed at 1 month (*p* < 0.001). Differences between groups were no longer statistically significant from 1 month onwards.

#### Macular volume (MV)

A similar pattern was observed for MV, which was higher at baseline in CRVO than in BRVO group. MV progressively declined over time in both subgroups, with no significant between-group differences after the first month.

**Table 2:** Longitudinal changes of vision and OCT parameters during treatment

Variables	Modeled mean (95%CI) ^f^			
	Total	CRVO	BRVO	*P* Value
**BCVA (logMAR)**				
At baseline^*0*^	0.79 (0.68, 0.90)	1.04 (0.87, 1.22)	0.64 (0.51, 0.77)	< 0.001*
At 1 month^1^	0.50 (0.39, 0.61)	0.63 (0.45, 0.80)	0.42 (0.29, 0.55)	0.066
At 3 months^3^	0.45 (0.35, 0.56)	0.56 (0.39, 0.74)	0.39 (0.26, 0.52)	0.107
At 6 months^6^	0.43 (0.32, 0.54)	0.56 (0.39, 0.73)	0.35 (0.22, 0.48)	0.056
At 12 months^12^	0.45 (0.34, 0.56)	0.55 (0.38, 0.72)	0.39 (0.26, 0.52)	0.150
*P* Value	*0–1*,* 0–3*,* 0–6*,* 0–12*, *P* < 0.001*	*0–1*,* 0–3*,* 0–6*,* 0–12*, *P* < 0.001*	*0–1*,* 0–3*,* 0–6*,* 0–12*, *P* < 0.001*	
**CRT**				
At baseline^*0*^	584.00 (555.23, 612.77)	688.52 (643.22, 733.81)	523.96 (489.63, 558.29)	< 0.001*
At 1 month^1^	294.63 (262.31, 326.96)	295.14 (239.96, 350.32)	291.67 (254.54, 328.79)	0.918
At 3 months^3^	287.27 (258.12, 316.42)	302.11 (256.82, 347.41)	278.72 (243.66, 313.79)	0.424
At 6 months^6^	321.33 (292.18, 350.48)	349.63 (304.34, 394.92)	304.82 (269.76, 339.88)	0.125
At 12 months^12^	297.45 (268.68, 326.21)	318.70 (273.41, 363.99)	285.23 (250.90, 319.56)	0.248
*P* Value	*0–1*,* 0–3*,* 0–6*,* 0–12*, *P* < 0.001*	*0–1*,* 0–3*,* 0–6*,* 0–12*, *P* < 0.001*	*0–1*,* 0–3*,* 0–6*,* 0–12*, *P* < 0.001*	
**MV**				
At baseline^*0*^	12.11 (11.75, 12.47)	13.25 (12.68, 13.82)	11.46 (11.02, 11.89)	< 0.001
At 1 month^1^	9.55 (9.15, 9.94)	10.05 (9.37, 10.72)	9.26 (8.80, 9.72)	0.060
At 3 months^3^	9.06 (8.70, 9.43)	9.35 (8.78, 9.92)	8.91 (8.46, 9.35)	0.230
At 6 months^6^	9.21 (8.85, 9.58)	9.64 (9.07, 10.21)	8.97 (8.53, 9.41)	0.070
At 12 months^12^	8.84 (8.48, 9.21)	9.25 (8.68, 9.83)	8.61 (8.17, 9.04)	0.079
*P* Value	*0–1*,* 0–3*,* 0–6*,* 0–12*, *P* < 0.001*	*0–1*,* 0–3*,* 0–6*,* 0–12*, *P* < 0.001*	*0–1*,* 0–3*,* 0–6*,* 0–12*, *P* < 0.001*	

CRVO, central retinal vein occlusion; BRVO, branch retinal vein occlusion; BCVA, best-corrected visual acuity; logMAR, logarithm of the minimal angle of resolution; CRT, central retinal thickness; MV, macular volume

^f^Derived from mixe- effect random intercept linear regression model

The longitudinal patterns of mean BCVA, CRT, MV values during follow-up were analyzed using a linear mixed model, in which the patients were considered as the random elements and time was the fixed effect

### Correlation between BCVA and retinal morphological parameters

Table 3 presents the correlations between CRT and MV with BCVA at various time points. Overall, logMAR BCVA showed a moderate positive correlation with the baseline CRT and MV. The correlation with MV remained significant at 1, 3, 6, and 12 months in contrast to that with CRT, which showed a significant correlation only at 1 and 12 months. In the CRVO group, BCVA was significantly correlated with CRT only at baseline, but correlated with MV at baseline, 1 and 12 months. In the BRVO group, the correlations between BCVA and CRT were significant at baseline, but not at other time points. The correlation with MV was marginally significant at baseline but did not reach statistical significance at subsequent time points.

**Table 3:** Strength and linear correlation of CRT, MV and BCVA at different timepoints determined by pearson’s correlation coefficient

BCVA	Parameters					
	Overall RVO		CRVO		BRVO	
	CRT	MV	CRT	MV	CRT	MV
Baseline	*r* = 0.49 *P* < 0.01*	*r* = 0.53 *P* < 0.01*	*r* = 0.44 *P* = 0.02*	*r* = 0.61 *P* < 0.01*	*r* = 0.39 *P* < 0.01*	*r* = 0.29 *P* = 0.05
1 month	*r* = 0.33 *P* = 0.01*	*r* = 0.39 *P* < 0.01*	*r* = 0.39 *P* = 0.07	*r* = 0.50 *P* = 0.02*	*r* = 0.10 *P* = 0.52	*r* = 0.10 *P* = 0.54
3 months	*r* = 0.23 *P* = 0.05	*r* = 0.28 *P* = 0.02*	*r* = 0.15 *P* = 0.45	*r* = 0.38 *P* = 0.05	*r* = 0.18 *P* = 0.22	*r* = 0.09 *P* = 0.55
6 months	*r* = 0.22 *P* = 0.06	*r* = 0.32 *P* = 0.01*	*r* = 0.13 *P* = 0.52	*r* = 0.33 *P* = 0.09	*r* = 0.14 *P* = 0.37	*r* = 0.14 *P* = 0.33
12 months	*r* = 0.26 *P* = 0.03*	*r* = 0.27 *P* = 0.02*	*r* = 0.23 *P* = 0.26	*r* = 0.40 *P* = 0.04*	*r* = 0.22 *P* = 0.13	*r* = 0.14 *P* = 0.36

CRVO, central retinal vein occlusion; BRVO, branch retinal vein occlusion; BCVA, best-corrected visual acuity; CRT, central retinal thickness; MV, macular volume

r = Pearson correlation coefficient

*Significant *P*-value

### Predictive relationship and causality of BCVA and morphological variability

The correlations between CRT-SD and MV-SD and BCVA at 12 months are shown in Table 4. In the linear regression analysis examining the association of macular thickness fluctuation and final VA at 12 months, both CRT-SD and MV-SD were found to have a significant correlation to VA. These findings suggest that greater CRT-SD and MV-SD were positively associated with worse visual outcomes at 12 months, although other factors not captured in the model may also influence visual acuity.

**Table 4:** Linear regression analysis between CRT-SD and MV-SD and BCVA at 12 months

Dependent Variable	Independent Variable	B coefficient	Standard error	95% CI for B coefficient	*R* ^2^	t statistic	*P* value
**Overall**							
VA at 12 months	CRT-SD	0.006	0.001	0.003 to 0.007	0.27	5.14	< 0.001
	MV-SD	0.386	0.110	0.168 to 0.605	0.14	3.52	< 0.001
**CRVO group**							
VA at 12 months	CRT-SD	0.004	0.001	0.002 to 0.006	0.32	3.45	0.002
	MV-SD	0.354	0.109	0.129 to 0.580	0.29	0.24	0.003
**BRVO group**							
VA at 12 months	CRT-SD	0.006	0.002	0.002 to 0.009	0.17	3.01	0.004
	MV-SD	0.436	0.192	0.050 to 0.822	0.10	2.28	0.028

CRVO, central retinal vein occlusion; BRVO, branch retinal vein occlusion; VA, visual acuity; CRT-SD, standard deviation of central retinal thickness; MV-SD, standard deviation of macular volume

R^2^ = coefficient of determination

## Discussion

This study demonstrated that both CRT and MV were significantly associated with final VA outcomes in patients with RVO treated with anti-VEGF agents. The correlation between the baseline CRT and MV and BCVA is consistent with the findings in previous studies that reported moderate associations between these parameters and visual outcomes in patients with RVO [6–10]. However, our study revealed that these correlations were more pronounced in the CRVO group than in the BRVO group. This disparity could be attributed to the more extensive retinal damage and subsequent higher fluid accumulation in CRVO, making CRT and MV more reflective of the disease severity and response to treatment [12]. Furthermore, BRVO is a uni-sectoral disease, and MV includes the unaffected side, therefore, it may have been difficult to obtain a correlation.

Our findings demonstrate a statistically significant association between CRT-SD and MV-SD at 12 months and visual acuity at the same time point. Greater fluctuations in macular thickness were associated with worse visual outcomes. These results are consistent with prior studies suggesting that morphologic instability of the macula may impair photoreceptor integrity and retinal function, ultimately limiting visual recovery [10, 13]. Although CRT-SD and MV-SD appeared to be a clinically relevant anatomical biomarker, the relatively modest R² highlights the multifactorial nature of visual prognosis in retinal diseases. Future studies should aim to elucidate the mechanisms underlying this relationship and explore its implications for patient management and prognosis.

Although our study confirmed the correlation value of CRT and MV, it also highlighted some limitations of these parameters. Specifically, relying solely on CRT for clinical decision-making might lead to premature treatment termination, as CRT does not always reflect changes in the parafoveal area. This is consistent with the findings of Fujihara-Mino et al. [7], who questioned the reliability of CRT as the sole marker for visual outcomes. Additionally, MV fluctuations were significantly correlated with changes in vision, suggesting that MV may be a more comprehensive marker for monitoring disease progression and response treatment.

The strengths of this study include the longitudinal 12-month follow-up, which provided robust data on the temporal relationship between OCT parameters and visual outcomes. Furthermore, the inclusion of only Asian patients provided valuable insights into RVO-induced changes in OCT parameters in this population, which has been underrepresented in previous studies. Nonetheless, this study had several limitations. The retrospective design and reliance on medical records may have introduced selection bias and limited the generalizability of the findings. Additionally, the objective of this study is to investigate the general association between OCT parameters and VA, rather than to specifically explore variations across ischemic and nonischemic RVO types. The use of various anti-VEGF agents with different injection protocols also adds variability to the treatment outcomes.

Future research should address these limitations by incorporating a prospective design with a standardized treatment protocol and a more balanced distribution of RVO subtypes. Including additional biometric parameters, such as presence of intraretinal or subretinal fluid, integrity of the ellipsoid layer, and disorganization of the inner retinal layers, would provide a more comprehensive assessment of macular health and its impact on visual outcomes. In BRVO patients, segmentation analysis of MV area may benefit more information. Moreover, expanding the study to include a diverse patient population would enhance the generalizability of the findings and contribute to the development of universally applicable treatment guidelines.

## Conclusions

This study highlighted the importance of concurrent CRT and MV evaluation during clinical assessment of patients with RVO undergoing anti-VEGF therapy. Although CRT and MV are both correlated with the final VA outcomes, MV yielded more correlation with visual outcomes in patients with RVO and ME receiving anti-VEGF therapy than CRT. Considering concurrent MV and CRT measurements could enhance more precision of treatment assessment, especially in CRVO patients. Future studies should focus on refining these predictive models and validating them across diverse patient populations to improve clinical decision-making and optimize patient care.

## Data Availability

No datasets were generated or analysed during the current study.

## Abbreviations

RVO: Retinal vein occlusion
BRVO: Branch retinal vein occlusion
CRVO: Central retinal vein occlusion
HRVO: Hemiretinal vein occlusion
ME: Macular edema
Anti-VEGF: Anti-vascular endothelial growth factor
OCT: Optical coherence tomography
CRT: Central retinal thickness
MV: Macular volume
BCVA: Best-corrected visual acuity
ICD-10: International classification of diseases, tenth revision
SD: Standard deviation
logMAR: Logarithm of the minimal angle of resolution
ETDRS: Early treatment diabetic retinopathy study
